# Beware the pancreatic incidentaloma in colorectal tumours: pancreatic adenocarcinoma with metastases to the colon and rectum

**DOI:** 10.1093/jscr/rjab629

**Published:** 2022-01-24

**Authors:** Brian O’Sullivan, Thomas Burton, Ralph Van Dalen, Fraser Welsh, Archana Pandita, Jesse Fischer

## Abstract

Colorectal cancer (CRC) is the third most diagnosed malignancy in the Western world. Routine staging of CRC often identifies incidental lesions on cross-sectional imaging. Appropriate treatment is dependent on a correct histological diagnosis. Pancreatic Ductal Adenocarcinoma (PDAC) is a rarer and often devastating diagnosis for which the treatment pathway differs significantly to CRC. We report two rare cases: the first recorded case of PDAC with synchronous rectal metastasis and a case of an acute presentation with large bowel obstruction from synchronous colonic metastasis. Both cases presented a significant diagnostic challenge. The management of both cases would have been altered had the histological diagnosis been known prior to surgery. Clinicians treating CRC should be wary of incidental lesions on staging investigations as they rarely represent an occult extra-intestinal primary malignancy. Immunohistochemistry plays an important role in ascertaining the origin of gastrointestinal malignancy.

## INTRODUCTION

Colorectal tumours are common and usually due to neoplastic transformation at various stages of the adenoma-carcinoma pathway [1]. Colorectal cancer (CRC) is the third most diagnosed malignancy in the Western world, with an estimated incidence of 38.7 per 100 000 persons per year [2]. Rectal adenocarcinoma accounts for almost one-third of all CRC [2]. Standard of care for rectal cancer includes neoadjuvant radiotherapy for locally advanced tumours (clinically T3 or above or lymph node positive). Histological confirmation of adenocarcinoma is recommended prior to treatment of CRC, but is particularly important when considering neoadjuvant therapy for rectal cancer [3]. Pancreatic ductal adenocarcinoma (PDAC) is a rarer but often devastating diagnosis; <20% of patients have surgically resectable disease at presentation and overall 5-year survival is ~5% [4]. Metastases to liver and lung are common and account for 90% of PDAC-associated deaths [5]. Metastatic disease to the gastrointestinal tract from PDAC is rare [6]. We report two cases of PDAC with synchronous colorectal metastases, each presenting a significant diagnostic challenge.

## CASE SERIES

### Case A

**Figure 1:**
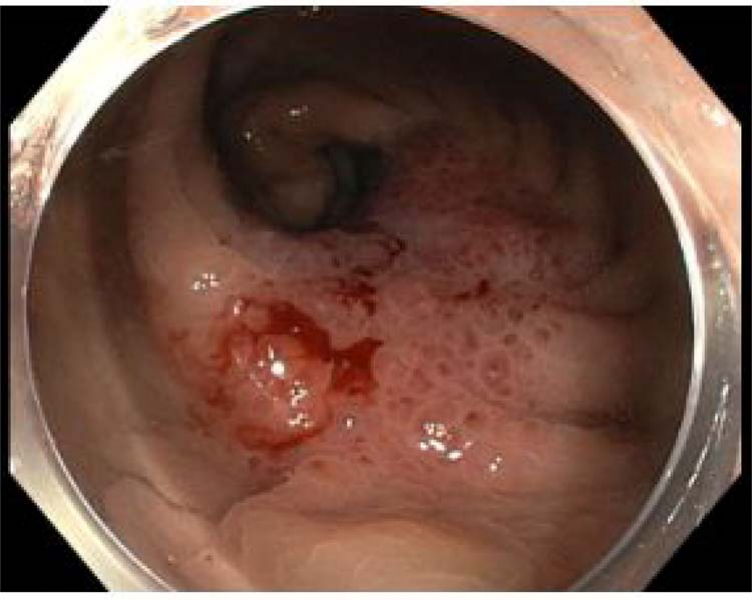
Case A. Endoscopic image at time of initial diagnosis with rectal tumour, prior to neoadjuvant chemoradiation.

An 82-year-old man with no significant medical history presented with progressive change in bowel habit and abdominal pain for 2 months. Colonoscopy 1 year prior had only revealed diverticulosis but on rectal examination at re-presentation a firm mass was palpable within the rectum. Colonoscopy was repeated and revealed an ulcerated non-obstructing low-rectal mass 3 cm in size (Fig. 1). Biopsies reported adenomatous change with at least high-grade dysplasia and a focus suspicious for invasive adenocarcinoma.

**Figure 2:**
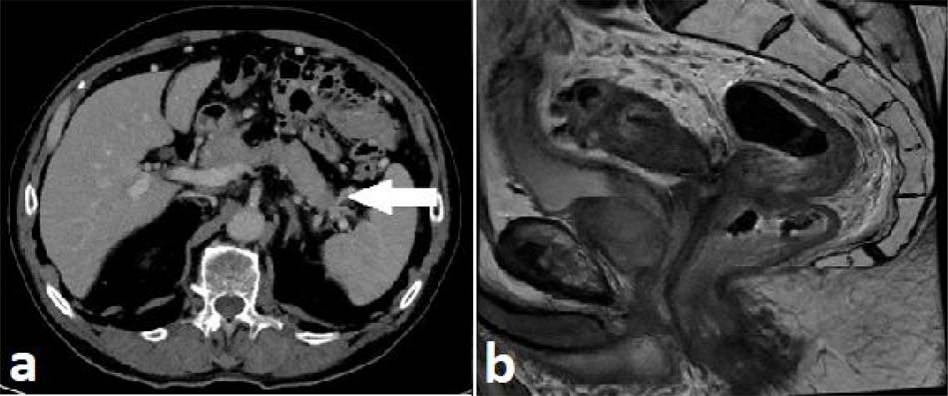
Case A. Axial portal venous phase CT scan demonstrating hypo-enhancing distal pancreatic lesion encasing splenic vessels (**a**) and sagittal MRI pelvis demonstrating anterior low rectal tumour (**b**), staged as a cT4aN2Mx rectal adenocarcinoma.

Routine staging for rectal cancer was performed. Magnetic resonance imaging (MRI) of the pelvis revealed an anterior rectal mass 7–8 cm from the anal verge with invasion into the seminal vesicles and at least three lymph nodes suspicious for metastases (cT4aN2). Computed tomography (CT) chest, abdomen and pelvis revealed enlarged nodes along the inferior mesenteric artery chain, but no definite metastatic disease was reported (Fig. 2). A 1.4 cm low attenuation hypo-enhancing mass in the distal pancreas occluding the splenic vein was reported as an incidental finding. Serum carcinoembryonic antigen (CEA) was 1.71ug/L and serum carbohydrate antigen 19-9 (Ca 19-9) was mildly elevated at 78u/ml.

Multidisciplinary team (MDT) discussion involving a pancreatic surgeon deemed a primary rectal adenocarcinoma with either incidental primary malignant or metastatic lesion in the distal pancreas as the likely diagnoses. Treatment recommended was neoadjuvant long-course chemoradiotherapy for the rectal tumour followed by rectal resection and distal pancreatectomy. Following neoadjuvant therapy restaging showed a slight reduction in size of pancreatic tumour from 14 to 12.6 mm and was again reported as consistent with a metastasis from a primary rectal malignancy. A laparoscopic ultra-low Hartmann’s procedure (gastrointestinal continuity not restored due to functional status preoperatively) combined with distal pancreatectomy and splenectomy was performed.

**Figure 3:**
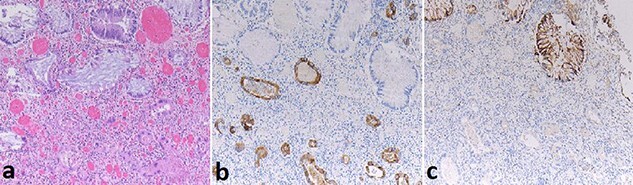
Case A. Specimen x10 magnification showing rectal metastasis from pancreatic ductal adenocarcinoma (**a**) with positive submucosal CK7+ immunostaining (**b**) and mucosal CK20+ immunostaining (**c**) but negative submucosal CK20 immunostaining(c).

Histopathology revealed a well to moderately differentiated, 18 mm PDAC in the pancreatic tail with lymphovascular and perineural invasion. The rectal specimen revealed a 32 mm mass in the anterior rectum and microscopically demonstrated grossly intact colorectal mucosa with extensive infiltration by adenocarcinoma from the sub-serosal aspect. The tumour in the submucosa and underlying structures of rectum showed immunoreactivity for CK7 while CK20 and CDX2 were negative; however, the overlying rectal mucosa showed reactivity to CK20 and CDX2 but was negative for CK7 (Fig. 3).

### Case B

**Figure 4:**
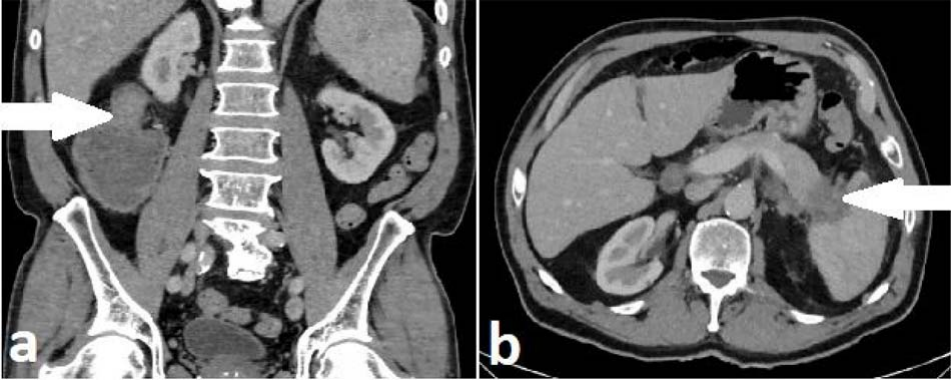
Case B. Portal venous phase CT scan of the abdomen. Coronal view of short segment hepatic flexure thickening (arrow) with proximal large bowel obstruction (**a**) and axial view of soft tissue mass (arrow) at tail of pancreas (**b**).

A 71-year-old man with a background of cardiac disease and obstructive sleep apnoea presented to the emergency department with symptoms of large bowel obstruction. Urgent CT abdomen and pelvis revealed short segment hepatic flexure thickening and stranding with evidence of obstruction at this point. An additional soft tissue mass in the tail of the pancreas encasing the splenic vasculature was reported as suspicious for metastatic disease from a primary colonic malignancy or a synchronous primary pancreatic malignancy (Fig. 4). Serum CEA was 5.32ug/L and serum Ca 19-9 was significantly elevated at 1180u/ml.

Due to the presence of large bowel obstruction, the patient proceeded to an urgent laparotomy. Operative findings were of a hard sclerotic mass within the pancreatic tail involving the splenic hilum. A further cicatrizing tumour was identified at the hepatic flexure with obstruction of the ascending colon. A right hemicolectomy and distal pancreatectomy with en-bloc splenectomy was performed.

Histopathology of the pancreatic resection revealed a 45 mm moderately differentiated PDAC with lymphovascular and perineural invasion. The colonic specimen revealed infiltration of metastatic PDAC from the serosal aspect into the muscularis and submucosa with focal areas infiltrating into mucosa. One of 18 mesocolic lymph nodes resected contained PDAC. Immunostaining of both tumours was positive for CK7 and negative for CK20.

## DISCUSSION

We report two cases of PDAC with synchronous colorectal metastases from the same institution within a 4-month period. In both instances a presumptive diagnosis of CRC with either distant metastasis to the pancreas or incidental pancreatic tumour was made, and treatment proceeded according to that recommended for CRC. If the true diagnosis was known the management of both cases could have differed significantly.

CRC is common and in a patient with lower gastrointestinal symptoms, imaging and/or histology suggestive of a primary CRC this is likely to be the presumed diagnosis, even in an MDT setting with numerous CRC specialists and a pancreatic surgeon. Pancreatic lesions are metastases in only 0.5–3% of cases [7] and pancreatic metastases from a colorectal primary are rare with only isolated case reports in the literature [8–10]. When distant CRC metastases are present, modern management tends to support multi-visceral resection if complete cytoreduction is possible [11], hence aggressive surgical treatment was pursued in both cases. The worldwide incidence of PDAC has more than doubled in the past 20 years [4] and will likely continue to grow with our ageing population. PDAC carries a poor prognosis and surgery is the only potentially curative treatment, but distant metastasis is a contra-indication to attempting curative resection [4, 5].

Metastasis of PDAC to the colon and rectum is extremely rare but may become more common with increasing incidence of PDAC. We describe the first reported case of PDAC with synchronous rectal metastasis, which was also occult. There is a single previous case report of PDAC metastasizing to the rectum [12], but this was a metachronous event in a patient with a history of pancreatic cancer resection and elevated pancreatic tumour markers. While the pancreatic lesion in case A was suspicious for malignancy, the possibility of a rectal metastasis from a PDAC was never strongly considered. The patient subsequently received routine treatment for locally advanced rectal cancer in our institution at the time; this treatment would not have been recommended had the diagnosis been known preoperatively and indeed non-operative management with palliative chemotherapy would usually be recommended for stage 4 PDAC. There have been six previous case reports of synchronous colonic metastases from PDAC [13–18], three reports of metachronous metastases from PDAC [12, 19, 20] and one report of metachronous colonic metastases from pancreatic acinar cell cancer (PACC) [21]. Pancreatic metastases were either confirmed or suspected preoperatively in three of those with synchronous disease [13, 16, 17] and two of those with metachronous disease [20, 21].

The rarity of colorectal metastasis from PDAC and lack of discerning imaging characteristics means radiological reporting is unlikely to suggest a pancreatic primary as the most likely diagnosis. Colonoscopy and biopsy should be performed when possible; the endoscopic appearance may not appear typical of a mucosal lesion such as CRC (Fig. 4). In case B, the presence of large bowel obstruction precluded colonoscopy; synchronous resection was performed due to concern post-operative recovery after right hemicolectomy could significantly delay elective pancreatic resection.

Along with the morphological histopathological features, immunohistochemistry strongly supported the diagnosis of PDAC metastasis following resection in both cases. Immunostaining was not performed on the diagnostic biopsy in Case A which showed tubular adenoma with a focus of high grade dysplasia; if only superficial rectal mucosal biopsies were obtained it may still have stained as for a CRC primary with immunohistochemistry. Histological diagnosis was not available preoperatively for Case B. Cytokeratin’s are intermediate filament proteins found in the intracytoplasmic skeleton of epithelial cells and are useful markers for epithelial malignant proliferations [22], CK7 immunostain is positive in upper gastrointestinal glandular epithelium and CK20 positive in lower gastrointestinal glandular epithelium. The colorectal specimens from both Case A and Case B show immunoreactivity for CK7 but not CK20 in the submucosal adenocarcinoma. In these clinical settings this finding is highly suggestive of metastatic PDAC and corresponds with the reported literature [12–20]. Mucosal dysplasia seen at diagnostic biopsy was not present in the resection specimen, and was presumed treated by chemoradiotherapy. Immunohistochemistry played an important vital role in determining the tumour tissue origin in both cases.

Case A showed mildly elevated serum CA19-9 and normal serum CEA levels. Two other case reports showed this trend, one being a synchronous metastasis [13] and the other a metachronous metastasis [19]. Case B showed elevated serum CA19-9 and minimally elevated serum CEA levels. Other cases had significant rises in both tumour markers [12, 14–16, 20], and while a high CA19-9 will alert the clinicians to the possibility of a PDAC, a low/normal CA19-9 does not exclude it. CEA may or may not be elevated in either diagnosis.

In conclusion, we report two rare cases of synchronous colorectal metastasis from PDAC in which the diagnosis was not known until after combined colorectal and pancreatic resection. The management of both cases, but case A (rectal metastasis) in particular, would have been significantly altered had the histological diagnosis been known prior to treatment. Immunohistochemistry is an important tool in determining the primary origin of a gastrointestinal malignancy and should be performed on pre-treatment biopsy where there is clinical possibility of an alternative primary site; this is particularly critical for rectal tumours where neoadjuvant therapy may be recommended.

## References

[1] Fearon ER, Vogelstein B. A genetic model for colorectal tumorigenesis. Cell 1990;61:759–67. 2188735 10.1016/0092-8674(90)90186-i

[2] Siegel R, Miller K, Goding Sauer A, Fedewa S, Butterly L, Anderson J, et al. Colorectal cancer statistics, 2020. CA Cancer J Clin 2020;70:145–64. 32133645 10.3322/caac.21601

[3] Hughet F, Muckerjee F, Javle M. Locally advanced pancreatic cancer: the role of definitive chemoradiotherapy. Clin Oncol 2014;26:560–8. 10.1016/j.clon.2014.06.002 25001636

[4] Pourshams A, Sepanlou S, Ikuta K, Bisignano C, Safiri S, Roshandel G, et al. The global, regional, and national burden of pancreatic cancer and its attributable risk factors in 195 countries and territories, 1990–2017: a systematic analysis for the global burden of disease study 2017. Lancet Gastroenterol Hepatol 2019;4:934–47. 31648972 10.1016/S2468-1253(19)30347-4 PMC7026711

[5] Das S, Batra S. Pancreatic cancer metastasis: are we being pre-EMTed? Curr Pharm Des 2015;21:1249–55. 25506899 10.2174/1381612821666141211115234 PMC4457289

[6] Cannistrà M, Ruggiero M, Zullo A, Serafini S, Grande R, Nardo B. Metastases of pancreatic adenocarcinoma: a systematic review of literature and a new functional concept. Int J Surg 2015;21:S15–S21. 10.1016/j.ijsu.2015.04.093 26123383

[7] Lasithiotakis K, Petrakis I, Georgiadis G, Paraskakis S, Chalkiadakis G, Chrysos E. Pancreatic resection for metastasis to the pancreas from colon and lung cancer, and osteosarcoma. JOP 2010;11:593–6. 21068492

[8] Lee CW, Wu RC, Hsu JT, Yeh CN, Yeh TS, Hwang TL, et al. Isolated pancreatic metastasis from rectal cancer: a case report and review of literature. World J Surg Oncol 2010;8:26. 20374636 10.1186/1477-7819-8-26 PMC2856583

[9] Tani R, Hori T, Yamada M, Yamamoto H, Harada H, Yamamoto M, et al. Metachronous pancreatic metastasis from rectal cancer that masqueraded as a primary pancreatic cancer: a rare and difficult-to-diagnose metastatic tumor in the pancreas. Am J Case Rep 2019;20:1781–7. 31784503 10.12659/AJCR.918669 PMC6910167

[10] Su L, Wernberg J. Synchronous distal pancreatic metastatic lesion arising from colonic adenocarcinoma: case report and literature review. Clin Med Res 2014;12:166–70. 24667222 10.3121/cmr.2013.1195 PMC4317154

[11] Stewart C, Warner S, Ito K, Raoof M, Wu G, Kessler J, et al. Cytoreduction for colorectal metastases: liver, lung, peritoneum, lymph nodes, bone, brain. When does it palliate, prolong survival, and potentially cure? Curr Probl Surg 2018;55:330–79. 30526930 10.1067/j.cpsurg.2018.08.004 PMC6422355

[12] Sun J, Zhang X, Huang H, Zhang Y, Wang K, Cui S. Rectal metastasis from a previously resected carcinoma of the pancreas: a case report. Transl Cancer Res 2020;9:3018–23. 10.21037/tcr.2020.02.74 PMC8798268 35117660

[13] Yewale R, Ramakrishna B, Vijaykumar K, Balasundaram P, Arulprakash S, Radhakrishna P, et al. Pancreatic adenocarcinoma with synchronous colonic metastases. ACG Case Reports Journal 2020;7:e00299. 32309493 10.14309/crj.0000000000000299 PMC7145160

[14] Bellows C, Gage T, Stark M, McCarty C, Haque S. Metastatic pancreatic carcinoma presenting as colon carcinoma. South Med J 2009;102:748–50. 19488001 10.1097/SMJ.0b013e3181a8fad7

[15] Kahl R, George K, Patel K, Stawick L. Pancreatic adenocarcinoma with rare sigmoid colon metastasis. ACG Case Reports Journal 2019;6:e00132. 31620529 10.14309/crj.0000000000000132 PMC6722371

[16] Nogueira S, Pinto B, Silva E, Garcia H, Carneiro F. Pancreatic cancer presenting as colonic disease. A rare case report. Int J Surg Case Rep 2018;44:4–7. 10.1016/j.ijscr.2018.01.019 PMC5852301 29454229

[17] Vrakas S, Kourkoulis P, Koutoufaris G, Manoloudaki K, Xourgias V. An unusual case of colonic mass. Clin Case Rep 2021;9:e04848. 34584713 10.1002/ccr3.4848 PMC8455961

[18] DY-Jung P, Krishnamurthi S, Chahal P, Downs-Kelly E, Morris-Stiff G. Pancreatic metastases to the colon: an unusual cause of colonic obstruction. BMJ Case Rep 2019;12:e228578. 10.1136/bcr-2018-228578 PMC6887360 31776144

[19] Inada K . Metachronous colonic metastasis from pancreatic cancer seven years post-pancreatoduodenectomy. World J Gastroenterol 2013;19:1665. 23539549 10.3748/wjg.v19.i10.1665 PMC3602487

[20] Kim W, Lee Y. Metachronous colonic metastasis from pancreatic cancer presenting as mechanical obstruction: a case report. Clin Imaging 2015;39:699–701. 25735449 10.1016/j.clinimag.2015.01.010

[21] Ohara Y, Oda T, Enomoto T, Hisakura K, Akashi Y, Ogawa K, et al. Surgical resection of hepatic and rectal metastases of pancreatic acinar cell carcinoma (PACC): a case report. World J Surg Oncol 2018;16:158. 10.1186/s12957-018-1457-8 PMC6091145 30075727

[22] Barak V, Goike H, Panaretakis K, Einarsson R. Clinical utility of cytokeratins as tumor markers. Clin Biochem 2004;37:529–40. 15234234 10.1016/j.clinbiochem.2004.05.009

